# T-Cell Response and Antibody Production Induced by the COVID-19 Booster Vaccine in Japanese Chronic Kidney Disease Patients Treated with Hemodialysis

**DOI:** 10.3390/vaccines11030653

**Published:** 2023-03-14

**Authors:** Ayumi Yoshifuji, Masataro Toda, Munekazu Ryuzaki, Emi Oyama, Kan Kikuchi, Toru Kawai, Ken Sakai, Masayoshi Koinuma, Kazuhiko Katayama, Takashi Yokoyama, Yuki Uehara, Norio Ohmagari, Yoshihiko Kanno, Hirofumi Kon, Toshio Shinoda, Yaoko Takano, Junko Tanaka, Kazuhiko Hora, Yasushi Nakazawa, Naoki Hasegawa, Norio Hanafusa, Fumihiko Hinoshita, Keita Morikane, Shu Wakino, Hidetomo Nakamoto, Yoshiaki Takemoto

**Affiliations:** 1 Infection Control Committee, The Japanese Society for Dialysis Therapy, Tokyo 113-0033, Japan; 2 Division of Nephrology, Department of Internal Medicine, Tokyo Saiseikai Central Hospital, Tokyo 108-0073, Japan; 3 Faculty of Pharmaceutical Sciences, Teikyo Heisei University, Tokyo 164-8530, Japan; 4 Laboratory of Viral Infection Control, Ōmura Satoshi Memorial Institute, Graduate School of Infection Control Sciences, Kitasato University, Tokyo 108-8641, Japan; 5 Department of Infectious Diseases, Keio University School of Medicine, Tokyo 160-8582, Japan

**Keywords:** COVID-19, hemodialysis, vaccination, vaccine, cellular immunity, humoral immunity, adverse reactions

## Abstract

Humoral and cellular responses are critical in understanding immune responses to severe acute respiratory syndrome coronavirus 2 (SARS-CoV-2) vaccination. Here, we evaluated these responses in hemodialysis (HD) patients after the booster vaccination. SARS-CoV-2 immunoglobulin (IgG) levels, neutralizing antibody titers, and the T-SPOT^®^.COVID test (T-SPOT) were measured prior to, three weeks after, and three months after the booster administration. The HD group had significantly higher SARS-CoV-2 IgG levels and neutralizing antibody titers against the original strain at three weeks and three months after the booster vaccination compared to the control group, albeit the HD group had lower SARS-CoV-2 IgG levels and neutralizing antibody titers before the booster administration. Moreover, the HD group had significantly higher T-SPOT levels at all three time points compared to the control group. The HD group also had significantly higher local and systemic adverse reaction rates than the control group. By booster vaccination, HD patients could acquire more effective SARS-CoV-2-specific humoral and cellular immunity than the control group.

## 1. Introduction

The coronavirus disease 2019 (COVID-19) pandemic caused by severe acute respiratory syndrome coronavirus 2 (SARS-CoV-2) has had devastating effects on healthcare, the economy, and society since 2020 [1]. Hemodialysis (HD) patients have more comorbidities and impaired immune function compared to healthy individuals, thus making them more susceptible to severe COVID-19; therefore, the mortality rate of COVID-19 is approximately 10 times higher among HD patients [2]. Vaccines are significant for the prevention of COVID-19 and its severe symptoms; therefore, nephrology societies have recommended that HD patients should be vaccinated [3]. However, a multivariable regression analysis showed that HD patients have lower antibody titers than healthy controls after the primary vaccine series (two-dose series) and identified a negative correlation between dialysis time (3–5 h) and antibody titers at 2 weeks after the primary series [4]. Moreover, the efficacy of the vaccine diminishes with time. During our study, the mean antibody titers of HD patients were 1085 BAU/mL at 2 weeks and 212.3 BAU/mL at 3 months after the primary series [4]. Therefore, booster doses are being administered worldwide. In Japan, the booster vaccine was first administered to healthcare workers in December 2021. In February 2022, individuals aged 18 years and older were eligible to receive the booster. In March 2022, individuals aged 12 years and older were eligible to receive the booster. As of September 2022, 65.4% of individuals of all ages and 90.5% of individuals aged 65 years and older had received the booster vaccine. In Japan, all eligible citizens are obligated by law to make an effort to receive the vaccine, and the government strongly recommends the booster vaccine [5]. Although higher antibody titers are associated with a lower risk of breakthrough infection and lower viral RNA copy numbers [6], there are no generally accepted clinical cutoff values for antibodies to protect against breakthrough infections or prevent severe disease. Because some studies have indicated that antibody titers are not strongly associated with the prevention of severe COVID-19 for HD patients [7], cellular immunity is critical for those who cannot achieve seroconversion [8,9]. Therefore, it is necessary to examine the efficacy of vaccines in terms of not only antibody titers but also cellular immunity. The T-SPOT^®^.COVID test (T-SPOT; Oxford Immunotec, Abingdon, UK), which is an enzyme-linked immunospot (ELISpot) assay, is a useful tool for evaluating the cellular immune response [10]. During this study, we evaluated the changes in the immune status of HD patients compared to the control, induced by the booster vaccine by measuring both antibody titers and cellular immunity.

## 2. Materials and Methods

### 2.1. Participants

We conducted a prospective, multicenter study after receiving approval from the Ethics Committee of the Japanese Society for Dialysis Therapy (approval number 1–10). Participants were enrolled from 6 July to 31 July 2021, using the Japanese Society for Dialysis Therapy website. Each dialysis facility recruited HD patients by posting information about this study in the hospital or clinic. The inclusion criteria for HD patients (HD group) were as follows: received the primary vaccine series (BNT162b2 vaccine); had not been infected with SARS-CoV-2; had not been treated for any malignancy within 1 year; had not been treated with drugs, such as steroids, immunosuppressants, and immunomodulators; were scheduled to receive a booster dose of the BNT162b2 vaccine; and had provided written consent for this study. The control group was registered by open recruitment at Tokyo Saiseikai Central Hospital and its affiliated facilities by matching the number of enrolled HD patients in terms of age (in 10-year increments) and sex. The control group also comprised patients who met the inclusion criteria for HD patients and had an estimated glomerular filtration rate ≥ 45 mL/min/1.73 m^2^.

### 2.2. Anti-S1 Antibody Titers

SARS-CoV-2 immunoglobulin (IgG) antibody titers of the S1 subunit of the spike protein of SARS-CoV-2 (anti-S1 antibody titers) were measured using the Ortho-Clinical Diagnostics VITROS^®^ anti-SARS-CoV-2 IgG chemiluminescent immunoassay and correlated with neutralizing antibodies at the following time points: 6 months (±1 week) after the primary series, 3 weeks (±3 days) after the booster dose, and 3 months (±1 week) after the booster dose. An antibody level ≥ 17.8 BAU/mL was considered positive. Patients with symptomatic COVID-19 during the study period were also excluded. Antibody titers over time were compared between groups. We defined the variation rate as the change in the anti-S1 antibody titer divided by the reference values, and we used the values of the anti-S1 antibody titer at 6 months after the primary series and 3 weeks after the third vaccine as the reference values. These were calculated as follows:

(Value at 3 weeks after the third vaccine–value at 6 months after the primary series)/value at 6 months after the primary series; and

(Value at 3 months after the third vaccine–value at 3 weeks after the third vaccine)/value at 3 weeks after the third vaccine.

### 2.3. Neutralizing Antibodies

The neutralizing antibodies were measured using VeroE6/transmembrane protease serine 2 (TMPRSS2) cells [11], which constitutively express TMPRSS2. The SARS-CoV-2 wild-type strain (original strain) (EPI_ISL_408667) provided by the National Institute of Infectious Diseases (Tokyo, Japan) and Omicron (B.1.1.529) variant strain clinically isolated at Keio University Hospital were prepared for the assay. Mutations in the two virus preparations used for the experiments were validated by whole genome sequencing [12]. The cells were maintained with Dulbecco’s modified Eagle medium with 5% fetal calf serum. The serum sample was serially diluted twice with Dulbecco’s modified Eagle medium, beginning with a 1:5 initial dilution, then it was mixed with an equal volume of 2 × 10^2^ median tissue culture infectious dose of SARS-CoV-2 and incubated at 37 °C for 1 h. Next, 100 µL of the mixture was mixed with an equal volume of VeroE6/TMPRSS2 cells (2 × 10^4^ cells/mL), incubated at 37 °C for 7 days, and monitored for cytopathic effects. The geometric mean titer of neutralizing antibodies was determined for each variant, along with the standard deviation. For calculations, the neutralizing antibody titer of the sample without any neutralization effect at a dilution of 1:10 was set as 5.

### 2.4. T-SPOT

The T-SPOT was performed to identify interferon (IFN)-γ-releasing T cells in response to stimulation with SARS-CoV-2 peptides because it is highly accurate (area under the curve, 0.95) in determining the presence of T cells specific to SARS-CoV-2, even several months after infection [10]. The T-SPOT was performed for participants between 50 and 80 years of age who provided consent for additional blood samples to be obtained. Specimens from hospitals that required more than 6 h for transportation to Tokyo Saiseikai Central Hospital were excluded because of specimen preservation issues. The T-SPOT was performed according to the manufacturer’s protocol. Peripheral blood samples were collected before treatment in heparinized tubes to isolate peripheral blood mononuclear cells. Lymphocytes were measured using a blood counting machine and adjusted to 2.5 × 10^5^/100 µL (acceptable range, 2.0 × 10^5^–3.0 × 10^5^). Isolated peripheral blood mononuclear cells were incubated with phytohemagglutinin in a microplate well as a positive control; additionally, isolated peripheral blood mononuclear cells were incubated with the medium in a microplate well as a negative control. The other wells contained SARS-CoV-2 peptides (CoV-A for spike protein and CoV-B for nucleocapsid protein). The microplates were incubated for 16 to 20 h at 37 °C with 5% CO_2_. An anti-IFN-γ antibody conjugate was added, and the number of spot-forming cells (SFCs) was counted using the ELISpot. Participants with 10 or more negative control SFCs and 20 or fewer positive control SFCs and those with eight or more CoV-B SFCs (previous infection) were excluded from this study. Individuals whose CoV-A minus negative control showed eight or more SFCs were diagnosed as positive. Individuals whose CoV-A minus negative control showed seven or fewer SFCs were diagnosed as negative. When quantifying and comparing the IFN-γ-releasing T cells in response to SARS-CoV-2, dividing by the median number of spots in response to the positive control can eliminate the impact of individual immune variations observed using the T-SPOT [13,14]. Therefore, we compared the results of dividing the number of SFCs in the CoV-A specimens by the number of SFCs in the phytohemagglutinin specimens between groups over time.

### 2.5. Adverse Reactions

A survey was conducted after the booster dose to investigate any adverse reactions (pain, redness, swelling, pruritus, fatigue, headache, muscle pain, coldness, fever [>37.5 °C], arthralgia, nausea, diarrhea, stomachache, and anaphylaxis), which were compared between groups. The questionnaire about adverse reactions is shown in Table S1.

### 2.6. Statistical Analysis

The SARS-CoV-2 IgG antibody was transformed into an ordinary logarithm and was analyzed by the unpaired *t*-test. T-SPOT was analyzed by the Mann–Whitney U test since the evaluation parameters were standardized values. Neutralizing antibody was also analyzed by the Mann–Whitney U test because the measured values were categorical. Frequencies between groups were compared using Fisher’s exact test or the χ^2^ test. Statistical significance was set at *p* < 0.05. Statistical analyses were performed using GraphPad Prism version 9.

## 3. Results

**Figure 1:**
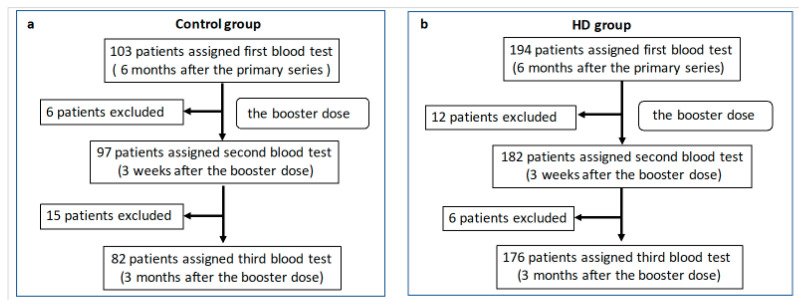
Trial profile. Blood samples were collected from 103 participants in the control group at 6 months after the primary series. Subsequently, one participant moved away, two were infected with severe acute respiratory syndrome coronavirus 2 (SARS-CoV-2), and three refused the booster dose. Therefore, blood samples were collected from the 97 remaining participants at 3 weeks after the booster dose. Thereafter, ten participants moved away or withdrew from the study, three were infected with SARS-CoV-2, and two could not be contacted. Therefore, blood samples were collected from the remaining 82 participants 3 months after the booster dose (**a**). Blood samples were collected from the 194 participants in the hemodialysis (HD) group at 6 months after the primary series. Subsequently, five participants were infected with SARS-CoV-2, two moved away, two died, and three refused booster vaccines. Therefore, blood samples were collected from the remaining 182 participants 3 weeks after the booster dose (3 weeks). Thereafter, four patients were hospitalized, one moved away, and one died. Therefore, blood samples were obtained from the 176 remaining participants at 3 months after the booster dose (3 months) (**b**).

Ten facilities (Tokyo Saiseikai Central Hospital, Harada Naika Clinic, Ozawa Clinic, Mizuno Clinic, Nakamura Clinic, Konan-no-sato, Shirogane-no-mori, Keifukuen, Oomori Nursing Home, and Kurara-Kaminoge) participated as the control group, and seven facilities (Shinagawa Dialysis Clinic, Meguro Station Building Clinic, Tokyo Saiseikai Central Hospital, Omiya Yoshizawa Clinic, Urawa Yoshizawa Clinic, Minami-Ooi Clinic, and Chuou Naika Clinic) participated as the HD group. The control group included a total of 103 participants, and the HD group included a total 194 participants (Figure 1).

### 3.1. HD Group Had Higher Anti-S1 Antibody Titers after Booster Administration than the Control

**Figure 2:**
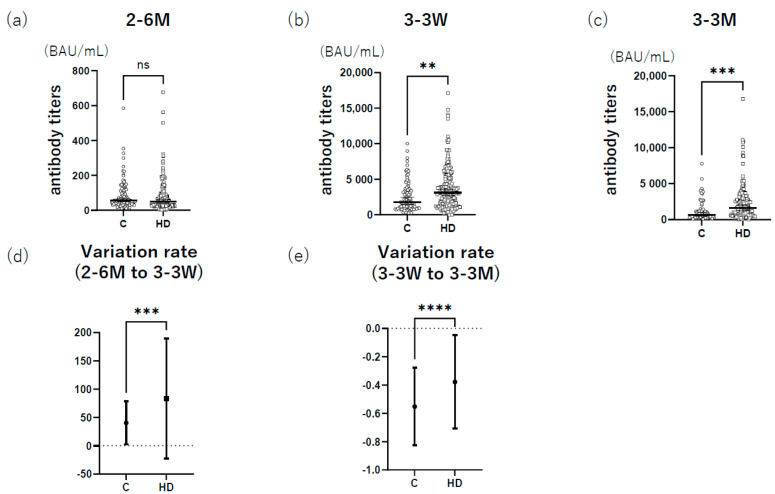
Comparison of antibody titers between the control and HD groups. Antibody titers in the control and HD groups were compared at three time points: (**a**) 6 months after the primary series (control group, *n* = 103; HD group, *n* = 194); (**b**) 3 weeks after the booster dose (control group, *n* = 97; HD group, *n* = 182); and (**c**) 3 months after the booster dose (control group, *n* = 82; HD group, *n* = 176). The variations from 6 months after the primary series to 3 weeks after the booster dose (**d**) and from 3 weeks after the booster dose to 3 months after the booster dose (**e**) were compared. Variations were calculated as follows: (**d**) (value at 3 weeks after the third vaccine–value at 6 months after the primary series)/value at 6 months after the primary series and (**e**) (value at 3 months after the third vaccine–value at 3 weeks after the third vaccine)/value at 3 weeks after the third vaccine. Bars indicate the median with a 95% confidence interval ** *p* ≤ 0.01, *** *p* ≤ 0.001, **** *p* ≤ 0.0001. 2–6 M: 6 months after the primary series; 3–3 W: 3 weeks after the booster dose; 3–3 M: 3 months after the booster dose; C: control; HD: Hemodialysis.

The characteristics of each group are shown in Table 1. Anti-S1 antibody titers at 6 months after the primary vaccine series in the HD group were not significantly different from those in the control group (*p* > 0.05) (Figure 2a). However, the HD group had significantly higher anti-S1 antibody titers at 3 weeks (*p* = 0.003) and 3 months after the booster dose (*p* = 0.0004) than the control group (Figure 2b,c). Additionally, the variation in anti-S1 antibody titers (from 6 months after the primary series to 3 weeks after the booster dose) was significantly higher in the HD group than in the control group (*p* < 0.0001) (Figure 2d). Furthermore, the variation in anti-S1 antibody titers (from 3 weeks after the booster dose to 3 months after the booster dose) was significantly lower in the HD group than in the control group (*p* < 0.0001) (Figure 2e). Twenty-nine participants withdrew from the study between blood collection at 6 months after the primary series and 3 months after the booster dose, which may have caused a selection bias. Therefore, antibody titers were re-evaluated only for those who completed blood collection until 3 months after the third dose, resulting in outcomes similar to those shown in Figure 2 (Figure S1).

### 3.2. HD Group Had Higher Neutralizing Antibodies after Booster Administration than the Control Group

Neutralizing antibodies of both the original and Omicron strains were evaluated. Regarding the original strain, neutralizing antibody levels were significantly lower in the HD group than in the control group at 6 months after the primary series (*p* = 0.01); however, they were significantly higher in the HD group than in the control group at 3 weeks and 3 months after the booster vaccine series (*p* < 0.0001 and *p* = 0.004, respectively). Neutralizing antibody levels of the Omicron strain showed trends similar to those of the original strain. Although there was no significant difference between the two groups at 6 months after the primary series and 3 weeks after the booster vaccine administration, the HD group had significantly higher levels than the control group at 3 months after the booster vaccine administration (*p* = 0.0001) (Table 2).

### 3.3. HD Group Had Higher T-SPOT Levels after Booster Administration than the Control

**Figure 3:**
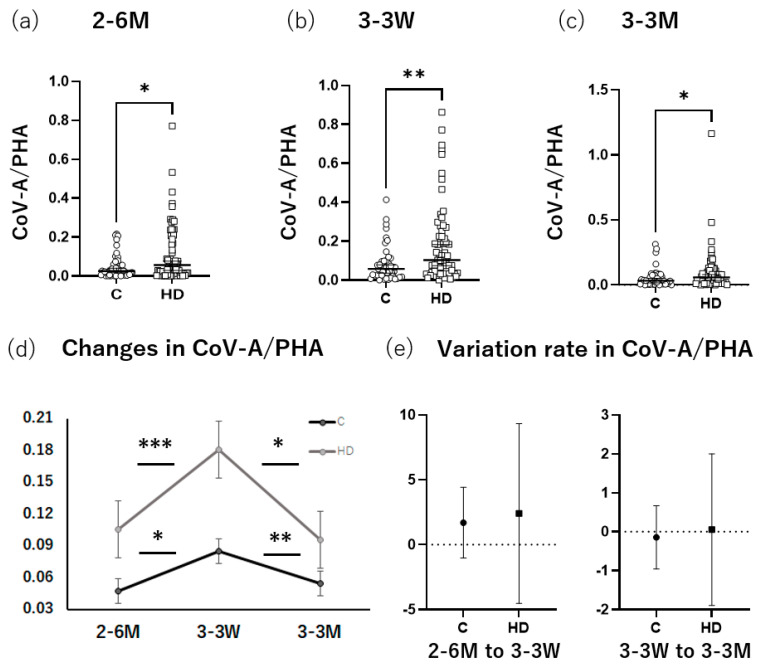
Changes in the T-cell response. The results of dividing the number of CoV-A spots found by the T-SPOT^®^.COVID test (T-SPOT) by the numbers of spots observed in the positive control (PHA) were compared between groups (control and HD) at three time points: (**a**) 6 months after the primary series; (**b**) 3 weeks after the booster dose; and (**c**) 3 months after the booster dose. For each group, the change in the CoV-A-to-PHA ratio found using the T-SPOT is shown (**d**). The values at 3 weeks after the booster dose were significantly higher than those at 6 months after the primary series and 3 months after the booster dose for both groups. The variation rate from 6 months after the primary series to 3 weeks after the booster dose and that from 3 weeks after the booster dose to 3 months after the booster dose was compared between the control and HD groups (**e**). No significant differences were detected. 2–6 M: 6 months after the primary series; 3–3 W: 3 weeks after the booster dose; 3–3 M: 3 months after the booster dose. C: control; HD: Hemodialysis. Bars indicate the median with a 95% confidence interval. * *p* ≤ 0.05, ** *p* ≤ 0.01, *** *p* ≤ 0.001. PHA, phytohemagglutinin.

Forty-eight participants were recruited as the control group (age, 63.4 ± 7.8 years; male, 66.7%) and 66 participants were recruited as the HD group (age, 69.2 ± 4.8 years; male, 57.8%) (Table S2). The results of the antibody titers at 6 months after the primary series and 3 weeks and 3 months after the booster dose were the same as those mentioned previously (Figure S2). The T-SPOT results were calculated by dividing the number of SFCs in the CoV-A specimen by the number of SFCs in the positive control to eliminate the effect of individual immune variability. Cases with a CoV-B spot count of eight or more were excluded as infection cases. As a result, the HD group had a significantly larger number of SFCs at 6 months after the primary series (*p* = 0.03), 3 weeks (*p* = 0.007) and 3 months after the booster dose (*p* = 0.04) than the control group (Figure 3a–c). In the control group, the number of SFCs at 3 weeks after the booster dose was higher than those at 6 months after the primary series (*p* = 0.02) and 3 months after the booster dose (*p* = 0.008). Additionally, in the HD group, the number of SFCs at 3 weeks after the booster dose was higher than those at 6 months after the primary series (*p* = 0.0004) and 3 months after the booster dose (*p* = 0.01) (Figure 3d). Although the variation rate from 6 months after the primary series to 3 weeks after the booster dose and that from 3 weeks after the booster dose to 3 months after the booster dose was compared between the control and HD groups (Figure 3e), no significant differences were detected. When the negative T-SPOT results were compared between the control and HD groups at 6 months after the primary series and 3 weeks and 3 months after the booster dose, the control group had a significantly higher rate of negative T-SPOT results than the HD group at 6 months after the primary series. At 3 weeks and 3 months after the booster dose, this rate tended to be higher in the control group than in the HD group; however, the difference was not significant (Table 3).

Table 4 shows the contingency table of positive and negative antibody levels and positive and negative T-SPOT results. Six months after the primary series, all patients with negative antibody levels had negative T-SPOT results in the control group. However, in the HD group, 57% (6.25%/10.94%) of patients with negative antibody levels had positive T-SPOT results. Additionally, at 3 weeks and 3 months after the booster dose, no patients had negative antibody levels in either group and the rate of negative T-SPOT results tended to be higher in the control group than the HD group but these were not significant.

### 3.4. HD Group Had Higher Local and Systemic Adverse Reaction Rates than the Control

Regarding the adverse reactions, the incidence rates of pruritus, fatigue, and muscle pain were significantly higher in the HD group (*p* = 0.008, *p* = 0.005, and *p* = 0.009, respectively) than in the control group after the booster dose. When these groups were further compared according to sex, the incidence rates of systemic adverse reactions (fatigue, muscle pain, coldness, and fever) were significantly higher in the HD group than in the control group (*p* = 0.04, *p* = 0.008, *p* = 0.004, and *p* = 0.001, respectively). In contrast, women in the HD group had significantly higher incidence rates of local adverse reactions (pain and pruritus) than women in the control group (*p* = 0.05 and *p* = 0.01, respectively) (Table 5).

## 4. Discussion

To investigate the effects of the BNT162b2 booster vaccine on HD patients, the immune responses, both cellular and humoral, were analyzed at 6 months after the primary series, 3 weeks after the booster dose, and 3 months after the booster dose. Although most studies of the effects of vaccines have focused on humoral immunity and antibody levels, this study analyzed the effects of vaccines on both humoral and cellular immunity. During our study, the antibody levels and neutralizing antibodies (original strain) in the HD group were higher than those in the control group at 3 weeks and 3 months after the booster dose, and the SARS-COV-2-specific T-cell responses found by the T-SPOT were significantly higher in the HD group than in the control group at 6 months after the primary series, and at 3 weeks, and 3 months after the booster dose.

HD patients had lower vaccine antibody titers, and these titers decreased rapidly with influenza and hepatitis B vaccines [15,16,17]. Antibody titers were significantly lower in the HD group than in the control group after the administration of the primary series of the BNT162b2 vaccine for COVID-19 [18,19]. Our previous study also showed that HD patients had significantly lower antibody levels than the controls up to 3 months after the administration of the primary series [4]. The results of the present study are contrary to our prediction of lower antibody levels in HD patients. Simon et al. conducted a similar study of the increase in antibody levels after booster vaccine administration. This study examined SARS-CoV-2-specific antibody titers of the control and HD groups at 6 to 8 weeks after the administration of the third vaccine and found no significant differences in antibody titers between the control and HD participants who achieved immunity responses. Although it is indicated that younger and female participants had a higher level of SARS-CoV-2-specific antibodies, the control group in the above study had younger and more female participants than the HD group [20]. Thus, the age-sex difference between the two groups caused an underestimation of antibody titers in the HD group. This study, which was age-sex matched, could evaluate the data more accurately, resulting in higher antibody titers and neutralizing antibodies (original strain) after the booster dose in the HD group than in the control group. No immunological studies have been conducted to determine why antibody titers are higher in HD patients than in the controls. Therefore, the reason for these results is unclear and further immunological studies are required.

Most studies have focused on antibodies as tools for humoral immunity. However, the T-cell response generally precedes the antibody response because of its necessity for priming B cells, and it is maintained for a longer period than the antibody response [21]. This has been demonstrated by studies that compared T-cell responses and antibody titers of patients with SARS-CoV-2. A strong T-cell response is possible even in the absence of antibody production [22]. Based on previous studies, this study also examined T-cell responses. Although T-cell responses after the primary series were not examined during our study, Clarke et al. reported that T-cell responses after the primary series were significantly lower in the HD group than in the control group [23]. This is consistent with the fact that end-stage kidney disease and uremia are associated with T-cell exhaustion and the suppression of IFN-γ production. However, Bernard et al. reported that T-cell responses after the primary series were equivalent in HD patients and controls [24]. Clarke et al. also noted that these differences may be attributable to differences in the SARS-CoV-2 peptides used for the ELISpot assay and the threshold used to define a positive result [23]. Our data at 6 months after the primary series showed that the T-SPOT levels were significantly higher and the negative T-SPOT results were significantly lower in the HD group than in the control group. The reasons for these findings remain unclear, but HD patients have a higher percentage of circulating T cells with interleukin-2 receptors; furthermore, these patients maintain high levels of plasma-soluble interleukin-2 receptors, resulting in a chronic preactivation state of T cells [25]. This may explain why the T-cell response was higher even at 6 months after the primary series. In the HD group, the originally activated T-cell response was further enhanced by the booster dose. Furthermore, at 3 weeks after the booster dose, the T-SPOT levels were still significantly higher in the HD group than in the control group, indicating that high cellular immunity was enhanced in the HD group. These results are similar to those reported by Bruminhent et al. [26], who examined the number of SARS-CoV-2-specific IFN-γ-producing T cells and S1 protein using the ELISpot assay (Mabtech, Cincinnati, OH, USA) and found that the numbers of IFN-γ-producing T cells after the booster dose were not significantly different between the control and HD groups. However, the median value was higher in the HD group after the booster dose than in the control group. Our study participants were age- and sex-matched, which may have caused this difference to be significant. In addition, only a few studies have compared antibody titers after the third vaccine in immunocompromised patients, such as those undergoing immunomodulating therapy or chemotherapy or those with lymphoproliferative diseases, and healthy controls. Although those immunocompromised patients showed a good antibody response, their antibody titers were lower than those of the healthy controls [27]. Therefore, HD patients and immunocompromised patients may have different immune responses to the booster dose.

Moreover, correlations between antibody levels and T-SPOT levels were examined during our study to investigate the relationship between cellular and humoral immunity. Previous studies of SARS-CoV-2 cases have shown that high antibody levels are correlated with high cellular immunity [28]. However, no correlation was observed during our study, and several HD patients with negative antibody levels also had T-cell responses, which may have an important role in the prevention of severe disease, as mentioned by McMahan et al. [8]. Both cellular and humoral immunity were significantly higher in the HD group than in the control group at 3 weeks and 3 months after the booster dose, which was considered helpful for the prevention of severe disease.

A comparison of the age-specific all-cause mortality rates of COVID-19 patients in the general population and HD patients in Japan from 6 January to 2 June 2022 (Omicron strain) showed that the mortality rates of the general population were 0.18%, 0.94%, and 3.36% for those in their sixth (age 60–69 years), seventh (age 70–79 years), and eighth (age 80–89 years) decades of life, respectively; however, those of HD patients in their sixth, seventh, and eighth decades of life were 2.40%, 4.30%, and 7.21%, respectively, indicating that the prognosis for HD patients remains worse than that for the general population. However, among HD patients who received the booster dose, the mortality rates were 0%, 1.14%, and 2.76% for those in their sixth, seventh, and eighth decades of life, respectively; these rates were similar to those of the general population [29,30]. As of late May 2022, 89.2% of individuals aged 65 years or older in the general population have received the booster. Our results may explain the reason that the mortality rate of HD patients has improved after the booster dose compared to that of the controls. However, because the innate immune system of HD patients is worse than that of the controls [31], higher cellular immunity and antibody levels may be necessary to protect these patients from infection.

The rates of systemic adverse reactions caused by the booster dose were significantly higher in the HD group than in the control group. We previously reported that systemic adverse reactions such as fever were worse in the HD group than in the control group after the primary series and that patients with stronger systemic adverse reactions had significantly higher antibody levels [4]. Yamamoto et al. reported that patients with stronger adverse reactions had higher antibody levels after the primary series and explained that the innate immune response after vaccination is related to the production of type I IFN, which triggers an adaptive immune response that includes the differentiation of T follicular helper cells and B cells into antibody-secreting plasma cells [32,33]. Type I IFNs and other cytokines caused systemic adverse reactions, including fever, leading to the activation of B cells generated by the first vaccine. Similarly, during this study, the stronger adverse reactions experienced by the HD patients compared to those of the controls may be related to the fact that the booster vaccine further enhanced the activation of B cells generated by the primary series because the antibody levels were higher in the HD group.

### Limitations

When recruiting patients, those younger than 65 years of age who were at risk for severe disease and those older than 65 years of age were targeted. The differences in the vaccination status among regions may have caused selection bias; however, efforts were made to reduce selection bias by recruiting patients from multiple facilities and regions. Furthermore, the differences in comorbidities, including diabetes mellitus, cerebrovascular disease, and cardiovascular disease, could have influenced the results of the comparisons between the control and HD groups. Additionally, the exclusion of symptomatic COVID-19 patients may have resulted in the selective elimination of individuals with a low immunity response who were more likely to have symptomatic COVID-19. Moreover, cellular immunity was evaluated only by the T-SPOT; no other cellular immunity analysis (such as other cytokine analyses) was performed. Further investigations of the mechanisms causing the antibody titer to be higher in the HD group after the booster dose and the T-cell response to be higher in the HD group than in the control group are required.

## 5. Conclusions

The HD group had significantly higher antibody titers, neutralizing antibodies (original strain), and SARS-CoV-2-specific T-cell responses at 3 weeks after the booster dose than the control group. The booster dose is an efficacious measure by inducing both humoral and cellular immunity to prevent severe cases of COVID-19 in HD patients.

## Figures and Tables

**Figure 4:**
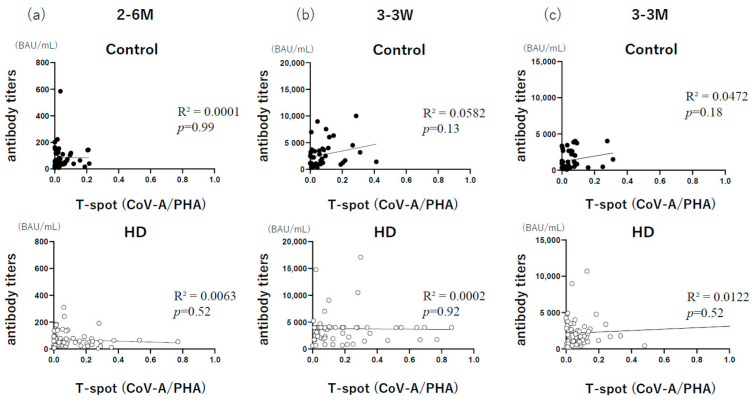
Correlation between the T-SPOT (CoV-A/PHA) results and antibody titers. The correlation between the T-SPOT COVID-19 results and antibody titers was examined at three-time points: (**a**) 6 months after the primary series, (**b**) 3 weeks after the booster dose, and (**c**) 3 months after the booster dose. 2–6 M, 6 months after the primary series; 3–3 W, 3 weeks after the booster dose; 3–3 M, 3 months after the booster dose. PHA, phytohemagglutinin.

**Table 1:** Characteristics of the participants.

	All			Males			Females		
	Control Group (*n* = 103)	HD Group (*n* = 194)	*p* Value	Control Group (*n* = 62)	HD Group (*n* = 126)	*p* Value	Control Group (*n* = 41)	HD Group (*n* = 68)	*p* Value
Males, *n* (%)	68 (61.8)	125 (64.8)	0.79						
Age, years (±SD)	65.4 ± 11.6	67.0 ± 11.1	0.31	64.8 ± 11.5	67.0 ± 11.5	0.78	66.5 ± 11.7	66.6 ± 10.2	0.78
BMI, kg/m^2^ (±SD)	23.6 ± 4.2	22.5 ± 4.3	0.03	24.1 ± 4.1	22.8 ± 3.9	0.01	22.8 ± 4.3	22.0 ± 4.8	0.01
Diabetes mellitus, *n* (%)	17 (16.3) *	79 (40.9)	<0.0001	13 (19.7) *	59 (47.2)	0.0002	4 (10.5) *	20 (29.4)	0.03
Hypertension, *n* (%)	48 (46.2) *	81 (42.0)	0.81	33 (50.0) *	55 (44.0)	0.54	15 (39.5) *	26 (38.2)	>0.99
Malignant tumor, *n* (%)	11 (10.6) *	29 (15.0)	0.29	8 (12.1) *	19 (15.2)	0.66	3 (7.9) *	10 (14.7)	0.37
Cerebrovascular disease, *n* (%)	3 (2.9) *	42 (21.8)	<0.0001	2 (3.0) *	31 (24.8)	0.0001	1 (2.6) *	11 (16.2)	0.052
Cardiovascular disease, *n* (%)	4 (3.8) *	37 (19.2)	<0.0001	4 (6.1) *	27 (21.6)	0.007	0 (0) *	10 (14.7)	0.013
COPD, *n* (%)	8 (7.7) *	11 (5.7)	0.63	5 (7.6) *	3 (2.4)	0.13	3 (7.9) *	8 (11.8)	0.74
Interval time ^†^ (±SD)	208 ± 18.9	213 ± 27.3	0.06	211 ± 18.9	216 ± 30.0	0.17	205 ± 18.3	209 ± 20.5	0.17
	* *n* = 104			* *n* = 66			* *n* = 38		

BMI: body mass index; COPD: chronic obstructive pulmonary disease; HD: hemodialysis; SD: standard deviation. * *n* = 107 because data from six patients were not available. ^†^ Interval time: days from the primary vaccine series to the booster vaccine. Age, BMI, and interval time are shown as average ± SD.

**Table 2:** Neutralizing antibodies.

2–6 M		Control	HD	*p* Value
Original	Mean ± SD	12.3 ± 3.1	9.0 ± 2.2	0.01
	95% CI	10.1–14.5	6.9–11.1	
Omicron	Mean ± SD	5.6 ± 1.7	5.1 ± 1.2	0.051
	95% CI	3.5–7.5	3.1–7.1	
**3–3 W**		**Control**	**HD**	***p* Value**
Original	Mean ± SD	324.7 ± 3.3	717.5 ± 3.4	<0.0001
	95% CI	322.5–326.9	715.4–719.6	
Omicron	Mean ± SD	43.8 ± 3.8	54.3 ± 3.2	0.18
	95% CI	38.1–42.5	51.9–56.1	
**3–3 M**		**Control**	**HD**	***p* Value**
Original	Mean ± SD	222.7 ± 3.4	365.6 ± 3.2	0.004
	95% CI	220.5–224.9	363.5–367.7	
Omicron	Mean ± SD	14.5 ± 3.2	26.7 ± 3.6	0.0001
	95% CI	11.0–15.4	24.1–28.5	

2–6 M: 6 months after the primary series; 3–3 W: 3 weeks after the booster dose; 3–3 M: 3 months after the booster dose. HD: Hemodialysis. 2–6 M: control group, *n* = 103; HD group, *n* = 194. 3–3 W: control group, *n* = 97; HD group, *n* = 182. 3–3 M: control group, *n* = 82; HD group, *n* = 176. SD: standard deviation, CI: confidence interval.

**Table 3:** Negative T-SPOT results for COVID-19.

	Control (%)	HD (%)	*p* Value
2–6 M	66.7 *	43.1 ^§^	0.02
3–3 W	35.0 ^†^	29.0 ^¶^	0.66
3–3 M	37.2 ^‡^	24.2 ^‖^	0.19

2–6 M: 6 months after the primary series. 3–3 W: 3 weeks after the booster dose. 3–3 M: 3 months after the booster dose. COVID-19: coronavirus disease 2019. HD: Hemodialysis. * *n* = 45; ^†^ *n* = 40; ^‡^ *n* = 43; ^§^ *n* = 65; ^¶^ *n* = 63; ^‖^ *n* = 62. The correlations between the T-SPOT results and antibody titers of each of the three measurement points in each group were evaluated (Figure 4). No significant correlations were observed at 6 months after the primary series, 3 weeks after the booster dose, or 3 months after the booster dose.

**Table 4:** Contingency table of T-SPOT results and SARS-CoV-2 IgG levels.

Control: 2–6 M				HD: 2–6 M			
		SARS-CoV-2 IgG				SARS-CoV-2 IgG	
		+ (%)	− (%)			+ (%)	− (%)
T-SPOT	+ (%)	34.78	0.00	T-SPOT	+ (%)	50.00	6.25
	− (%)	54.35	10.87		− (%)	39.06	4.69
**Control: 3–3 W**				**HD: 3–3 W**			
		SARS-CoV-2 IgG				SARS-CoV-2 IgG	
		+ (%)	− (%)			+ (%)	− (%)
T-SPOT	+ (%)	65.00	0.00	T-SPOT	+ (%)	70.97	0.00
	− (%)	35.00	0.00		− (%)	29.03	0.00
**Control: 3–3 M**				**HD: 3–3 M**			
		SARS-CoV-2 IgG				SARS-CoV-2 IgG	
		+ (%)	− (%)			+ (%)	− (%)
T-SPOT	+ (%)	62.79	0.00	T-SPOT	+ (%)	75.81	0.00
	− (%)	37.21	0.00		− (%)	24.19	0.00

2–6 M, 6 months after the primary series; 3–3 W, 3 weeks after the booster dose; 3–3 M, 3 months after the booster dose. T-SPOT positive (+): ≥8 CoV-A-negative control spots; T-SPOT negative (−): ≤7 CoV-A-negative control spots; SARS-CoV-2 IgG-positive (+): ≥17.8 BAU/mL; SARS-CoV-2 IgG-negative (−): <17.8 BAU/mL. IgG: immunoglobulin.

**Table 5:** Adverse reactions after vaccination.

All														
	Local *n* (%)				Systemic *n* (%)									
	Pain	Redness	Swelling	Pruritus	Fatigue	Headache	Muscle Pain	Coldness	Fever	Arthralgia	Nausea	Diarrhea	Stomachache	Anaphylaxis
Control (*n* = 87)	42(48.3)	7(8.0)	5(5.7)	5(5.7)	11(12.6)	6(6.9)	9(10.3)	6(6.9)	10(11.5)	3(3.4)	0(0)	2(2.3)	0(0)	0(0)
HD (*n* = 184)	104(56.5)	18(9.8)	24(13.0)	33(17.9)	52(28.3)	18(9.8)	44(23.9)	28(15.2)	45(24.5)	20(10.9)	7(3.8)	2(1.1)	5(2.7)	0(0)
*p*-value	0.24	0.82	0.09	0.008	0.005	0.5	0.009	0.08	0.015	0.06	0.1	0.59	0.18	>0.99
**Male**														
	**Local *n* (%)**				**Systemic *n* (%)**									
	**Pain**	**Redness**	**Swelling**	**Pruritus**	**Fatigue**	**Headache**	**Muscle Pain**	**Coldness**	**Fever**	**Arthralgia**	**Nausea**	**Diarrhea**	**Stomachache**	**Anaphylaxis**
Control (*n* = 51)	23(45.1)	1(0.2)	1(0.2)	1(0.2)	5(9.8)	1(0.2)	3(5.9)	0(0)	2(3.9)	1(0.2)	0(0)	0(0)	0(0)	0(0)
HD (*n* = 121)	57(47.1)	9(7.4)	11(9.1)	11(9.1)	30(24.8)	6(5.0)	27(22.3)	17(14.0)	29(24.0)	11(9.1)	4(3.3)	2(1.7)	3(2.5)	0(0)
*p*-value	0.87	0.29	0.11	0.11	0.04	0.68	0.008	0.004	0.001	0.11	0.32	>0.99	0.56	>0.99
**Female**														
	**Local *n* (%)**				**Systemic *n* (%)**									
	**Pain**	**Redness**	**Swelling**	**Pruritus**	**Fatigue**	**Headache**	**Muscle Pain**	**Coldness**	**Fever**	**Arthralgia**	**Nausea**	**Diarrhea**	**Stomachache**	**Anaphylaxis**
Control (*n* = 36)	19(52.8)	6(16.7)	4(11.1)	4(11.1)	6(16.7)	5(13.9)	6(16.7)	6(16.7)	8(22.2)	2(5.6)	0(0)	2(5.6)	0(0)	0(0)
HD (*n* = 63)	47(74.6)	10(15.9)	13(20.6)	22(34.9)	22(34.9)	12(19.0)	17(27.0)	11(17.5)	16(25.4)	9(14.3)	3(4.8)	0(0)	2(3.2)	0(0)
*p*-value	0.05	>0.99	0.28	0.01	0.07	0.59	0.32	>0.99	0.81	0.32	0.55	0.13	0.53	>0.99

HD; hemodialysis.

## Data Availability

The datasets generated and analyzed during this study are available from the corresponding author on reasonable request.

## Supplementary Materials

The following supporting information can be downloaded at: https://www.mdpi.com/article/10.3390/vaccines11030653/s1, Figure S1: Comparison of antibody titers between the control and HD groups (only for participants who participated in all blood tests); Figure S2: Comparison of neutralizing antibody titers between the control and HD groups; Figure S3: Comparison of antibody titers of the participants in the control and HD groups evaluated using the T-SPOT^®^.COVID test (T-SPOT); Table S1: Questionnaire about adverse reactions after the booster vaccination; Table S2: Characteristics of participants in the T-SPOT study.
