# Supplementary material for: T-Cell Response and Antibody Production Induced by the COVID-19 Booster Vaccine in Japanese Chronic Kidney Disease Patients Treated with Hemodialysis
Source: Vaccines (Basel). 2023 Mar 14;11(3):653. doi: 10.3390/vaccines11030653 (PMC10057502; doi:10.3390/vaccines11030653)
Supplement: Supplementary file 1 [file vaccines-11-00653-s001.zip › vaccines-2194445-supplementary.pdf]

vaccines MDPI

Supplementary Materials

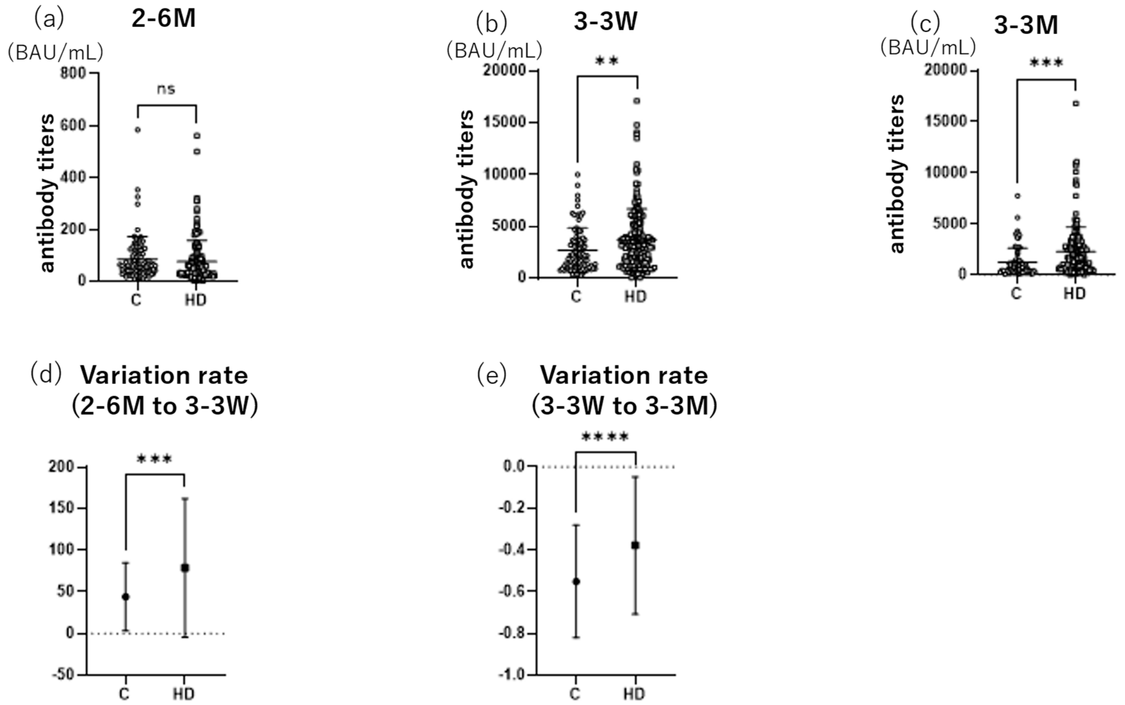

Figure S1. Comparison of antibody titers between the control and HD groups (only for participants who participated in all blood tests). At 6 months after the primary series, blood was obtained from 297 participants (control group, n=103; HD group, n=194); however, 29 participants withdrew from the study at 3 months after the booster dose. Blood was obtained from 258 participants (control group, n=82; HD group, n=176) at 3 months after the booster dose. (a) 6 months after the primary series. (b) 3 weeks after the booster dose. (c) 3 months after the booster dose. (d) Value at 3 weeks after the third vaccine - value at 6 months after the primary series/value at 6 months after the primary series. (e) Value at 3 months after the third vaccine – value at 3 weeks after the third vaccine/value at 3 weeks after the third vaccine. Bars indicate the median with a 95% confidence interval. $^{*}P\leq0.05$, $^{**}P\leq0.01$, $^{***}P\leq0.001$, $^{****}P\leq 0.0001$. C: control; HD: hemodialysis. 2-6M: 6 months after the primary series; 3-3W: 3 weeks after the booster dose; 3-3M: 3 months after the booster dose.

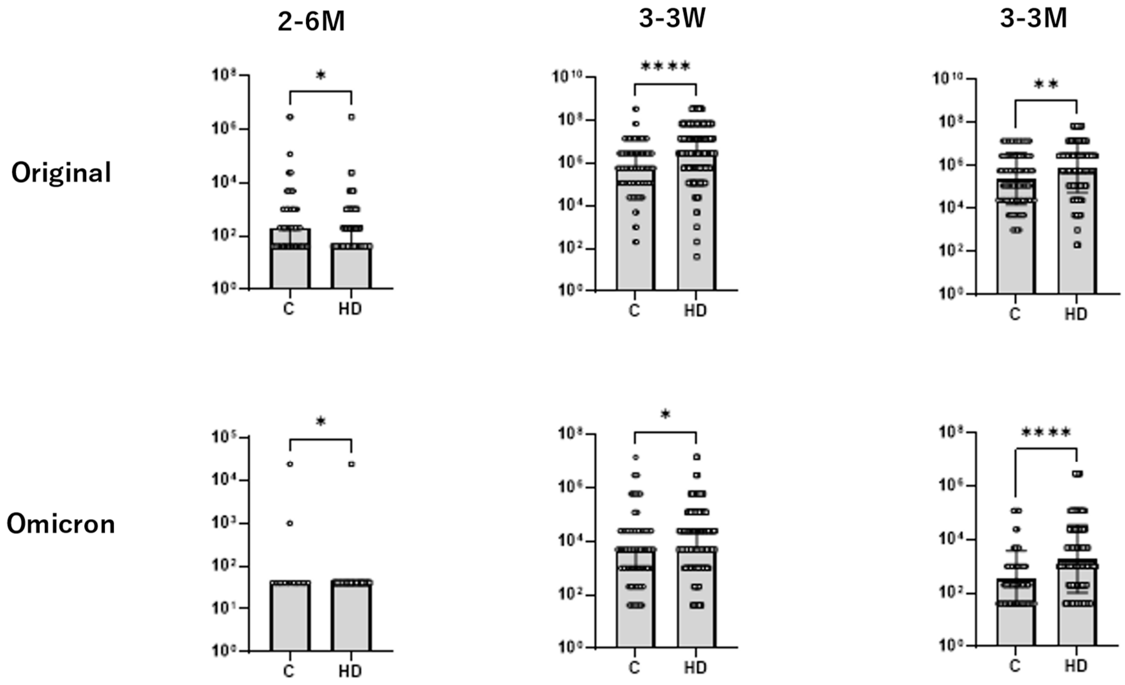

Figure S2. Comparison of neutralizing antibody titers between the control and HD groups. Neutralizing antibody titers in the control and HD groups were compared at three-time points: (a) 6 months after the primary series (control group, n=103; HD group, n=194); (b) 3 weeks after the booster dose (control group, n=97; HD group, n=182); and (c) 3 months after the booster dose (control group, n=82; HD group, n=176). Bars indicate the median with a 95% confidence interval *p≤0.05, **p≤0.01, ***p≤0.001, ****p≤0.0001. 2-6M: 6 months after the primary series; 3-3W: 3 weeks after the booster dose; 3-3M: 3 months after the booster dose; C: control; HD: Hemodialysis.

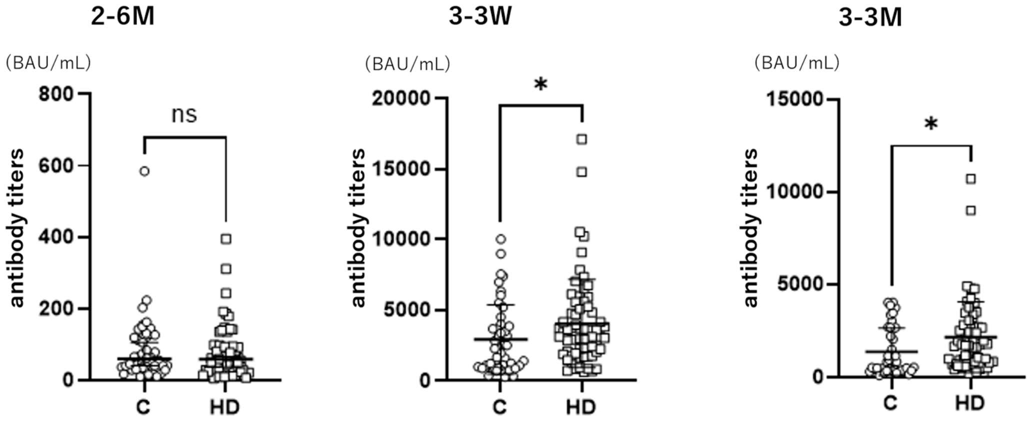

Figure S3. Comparison of antibody titers of the participants in the control and HD groups evaluated using the T-SPOT®.COVID test (T-SPOT). The severe acute respiratory syndrome coronavirus 2

(SARS-CoV-2) immunoglobulin (IgG) levels of the control and HD groups were compared. (a) 6 months after the primary series. (b) 3 weeks after the booster dose. (c) 3 months after the booster dose. Bars indicate the median with a 95% confidence interval *P≤0.05. C: control; HD: hemodialysis. 2-6M, 6 months after the primary series; 3-3W, 3 weeks after the booster dose; 3-3M, 3 months after the booster dose.

Table S1. Questionnaire about adverse reactions after the booster vaccination

**Questionnaire code -**	
**Questionnaire on adverse reactions to booster vaccination.**	
I received the booster dose of the vaccine on date _____month _____ 2021.	
Please answer the following questions about adverse reactions to the booster vaccination.	
1. Did you have any pain at the injection site?	Yes (lasted for ____ days) / No
2. Did you experience redness at the injection site?	Yes (lasted for ____ days) / No
3. Did you experience swelling at the injection site?	Yes (lasted for ____ days) / No
4. Did the injection site become itchy?	Yes (lasted for ____ days) / No
5. Did you feel tired all over your body?	Yes (lasted for ____ days) / No
6. Do you have headache?	Yes (lasted for ____ days) / No
7. Do you have muscle pain?	Yes (lasted for ____ days) / No
8. Do you feel chilly?	Yes (lasted for ____ days) / No
9. Did you have fever?	Yes (max. °C, lasted for days) / No
10. Did you have any joint pain?	Yes (lasted for ____ days) / No
11. Did you have nausea or vomiting?	Yes (lasted for ____ days) / No
12. Do you have diarrhea?	Yes (lasted for ____ days) / No
13. Do you have stomachache?	Yes (lasted for ____ days) / No
14. Did you have allergic symptoms or anaphylaxis? Please select from the following.	
A: Allergic symptoms occurred (please specify:)	
B: Anaphylaxis occurred (please specify:)	
C: I was able to receive the inoculation without any particular problem.	
Thank you very much for your cooperation.	

Table S2. Characteristics of participants in the T-SPOT study.

	**Control group (n=48)**	HD group (n=64)	*p* value
Male, n (%)	31 (64.6)	35 (54.7)	0.2
Age, years (±SD)	62.9±7.9	69.4±4.8	**0.02**
BMI, kg/m^2 (±SD)	24.1±4.8	22.0±4.6	**0.02**
Diabetes mellitus, n (%)	9 (18.8)	21 (32.8)	0.07
Hypertension, n (%)	25 (52.1)	22 (34.4)	0.05
Malignant tumor, n (%)	6 (12.5)	13 (20.3)	0.2
Cerebrovascular disease, n (%)	7 (14.6)	14 (21.9)	0.23
Cardiovascular disease, n (%)	7 (14.6)	11 (17.2)	0.5
COPD, n (%)	3 (6.3)	4 (6.3)	0.65
Interval time* average ± SD	208.9±15.1	215±16.9	**0.03**

BMI: body mass index; COPD: chronic obstructive pulmonary disease; HD: hemodialysis; SD: standard deviation. *Interval time: days from primary series to booster vaccination.
